# Supplementary material for: The interactome of intact mitochondria by cross-linking mass spectrometry provides evidence for coexisting respiratory supercomplexes
Source: Mol Cell Proteomics. 2017 Dec 8;17(2):216–32. doi: 10.1074/mcp.RA117.000470 (PMC5795388; doi:10.1074/mcp.RA117.000470)
Supplement: Supplemental Data [file supp_RA117.000470_133922_0_supp_23978_fzffwf.zip › spectra_annotation/mito_spectra_annotation/409-1-2-1-9-1.pdf]

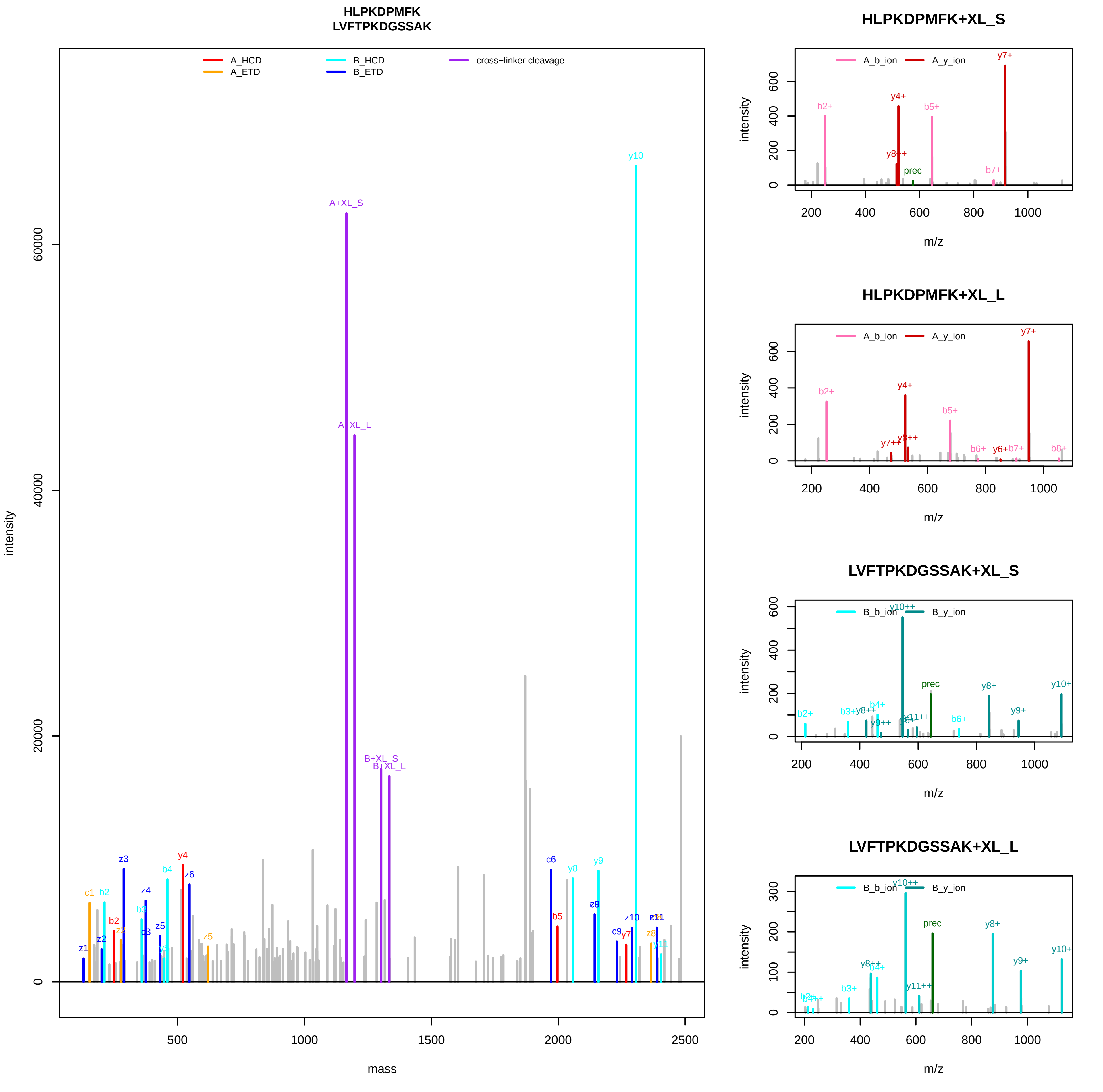
HLPKDPMFK
LVFTPKDGSSAK
A_HCD
A_ETD
B_HCD
B_ETD
cross−linker cleavage
intensity
mass
0
20000
40000
60000
500
1000
1500
2000
2500
y10
A+XL_S
A+XL_L
B+XL_S
B+XL_L
z1
c1
b2
z2
b2
z2
z3
b3
c3
z4
z5
b4
y4
z6
z5
c6
b5
y8
y9
z9
c9
y7
z10
z8
z11
y11
HLPKDPMFK+XL_S
HLPKDPMFK+XL_L
LVFTPKDGSSAK+XL_S
LVFTPKDGSSAK+XL_L
A_b_ion
A_y_ion
B_b_ion
B_y_ion
m/z
prec
b2+
y4+
b5+
y7+
y8++
b7+
y7++
b6+
y6+
b8+
y10++
y8+
y9+
y10+
b3+
b4+
y9++
y11++
b6+
